# PAR-1 signaling on macrophages is required for effective in vivo delayed-type hypersensitivity responses

**DOI:** 10.1016/j.isci.2020.101981

**Published:** 2021-01-05

**Authors:** Hannah Wilkinson, Hugh Leonard, Daxin Chen, Toby Lawrence, Michael Robson, Pieter. Goossens, John H. McVey, Anthony Dorling

**Affiliations:** 1 Department of Inflammation Biology, School of Immunology & Microbial Sciences, King's College London, Guy's Hospital, London SE1 9RT, UK; 2 Centre for Inflammation Biology and Cancer Immunology, School of Immunology & Microbial Sciences, King's College London, London SE1 9RT, UK; 3 Department of Pathology, Cardiovascular Research Institute Maastricht (CARIM), Maastricht University, 6229HX Maastricht, the Netherlands; 4 School of Bioscience & Medicine, Faculty of Health and Medical Sciences, University of Surrey, Guildford GU2 7XH, UK

**Keywords:** Molecular Biology, Immunology

## Abstract

Delayed-type hypersensitivity (DTH) responses underpin chronic inflammation. Using a model of oxazolone-induced dermatitis and a combination of transgenic mice, adoptive cell transfer, and selective agonists/antagonists against protease activated receptors, we show that that PAR-1 signaling on macrophages by thrombin is required for effective granuloma formation. Using BM-derived macrophages (BMMs) *in vitro*, we show that thrombin signaling induced (a) downregulation of cell membrane reverse cholesterol transporter ABCA1 and (b) increased expression of IFNγ receptor and enhanced co-localization within increased areas of cholesterol-rich membrane microdomains. These two key phenotypic changes combined to make thrombin-primed BMMs sensitive to M1 polarization by 1000-fold less IFNγ, compared to resting BMMs. We confirm that changes in ABCA1 expression were directly responsible for the exquisite sensitivity to IFNγ *in vitro* and for the impact on granuloma formation *in vivo*. These data indicate that PAR-1 signaling plays a hitherto unrecognized and critical role in DTH responses.

## Highlights

• Inhibiting thrombin signaling on monocytes is protective in type IV hypersensitivity
• PTL060, a thrombin inhibitor, reduces inflammation in type IV hypersensitivity
• Thrombin primes macrophages to be highly sensitive to IFNγ and LPS
• Thrombin increases lipid rafts on macrophages in an ABCA1-dependent manner

## Graphical Abstract

**Figure:**
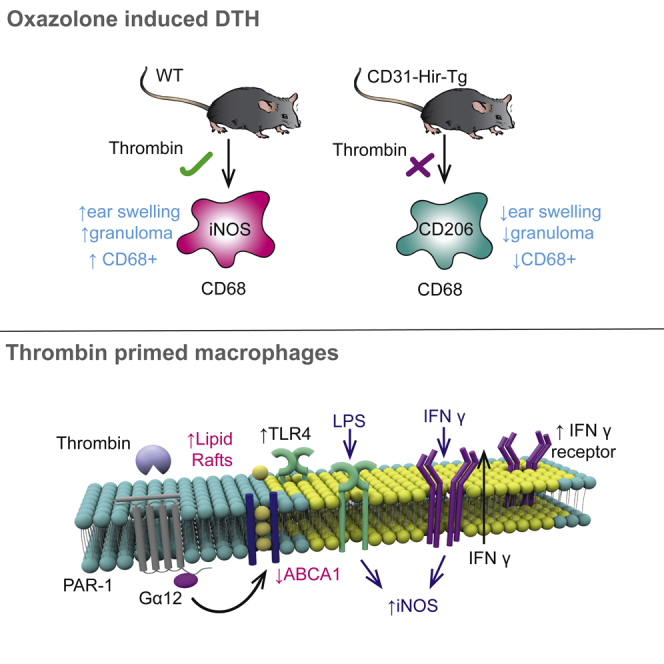

## Introduction

Macrophages are heterogeneous and versatile cells found in virtually all tissues of adult mammals. Activation of macrophages has emerged as a key area of immunology, tissue homeostasis, disease pathogenesis, and resolving and non-resolving inflammation. Early literature described them dichotomously as M1 or M2 macrophages (Mills, 2015), with M1 macrophages being the classical inflammatory macrophages induced by T-cell-dependent (interferon γ [IFNγ]) and T-cell-independent (lipopolysaccharide [LPS]) pathways. These promote upregulation of Th1 proinflammatory chemokines and cytokines such as IL-6, IL-12, and IL-23. They upregulate HLA-DR, thus having a role in antigen presentation and induce nitric oxide production. In contrast to M1 macrophages, M2 macrophages are anti-inflammatory having roles in tissue homeostasis and repair and have roles in the Th2 response. M2 macrophages are classically induced by IL-4 or IL-13. As time has progressed, these two are recognized as extreme phenotypes, with subtypes described *in vivo* appearing more plastic and often expressing characteristics of both. Subsets with a predominant M2 phenotype (M2a-d) have been defined, having anti-inflammatory roles in the Th2 response (M2a), suppression of tumor growth (M2b), immune regulation and tissue remodeling (M2c), and angiogenesis (M2d). These M2 subsets have different polarizing stimuli, eg. IL-4/13 – M2a, immune complexes, and toll-like receptor (TLR) ligands – M2b, IL-10 & TGF- beta- M2c and IL-6 for M2d macrophages. Further subsets have been defined in the field of atherosclerosis research (Adamson and Leitinger, 2011) including further anti-inflammatory atheroprotective subtypes M(Hb) M(heam) and Mox (Moore et al., 2013). What is becoming clear is the classical/alternative model of macrophage activation does not take into account the subtle changes occurring in the cell microenvironment which can have tangible changes to the cell phenotype without fully polarizing the cells.

Type IV or delayed-type hypersensitivity (DTH) is the archetypal antigen-specific cell-mediated immune response involving CD4+ T cells and monocytes/macrophages. In the sensitization phase, antigen-presenting cells present the hapten (e.g., oxazolone) to naive T cells. The T cells then expand to a group of hapten-specific T-helper1 (TH1) cells. In the effector phase, re-challenge with the same hapten leads to rapid expansion of the of the sensitized TH1 cells which then interact with resting macrophages leading to macrophage activation via IFNγ and tumor necrosis factor beta (TGF-β) (Chen et al., 2019). These interactions underpin the chronic inflammatory lesions characteristic of inflammatory bowel disease, chronic infection, sarcoidosis, and rejection of transplanted kidneys (Black, 1999).

Thrombin is a serine protease generated at the site of tissue injury and is the main effector enzyme in the coagulation cascade (Coughlin, 2005; Shrivastava et al., 2013). Thrombin generation is initiated by tissue factor (TF), a transmembrane protein found on the adventitia of vessels, as well as on tissue macrophages, dendritic cells, and at low levels on circulating monocytes. In addition to the well-described role of thrombin in coagulation, it has a direct effect on a wide array of cell types such as smooth muscle cells, platelets, and endothelial cells (ECs) (Cunningham et al., 2000). These cellular responses of thrombin are mediated through a family of G-protein-coupled protease activated receptors (PARs), designated PAR-1-4 (Coughlin, 2005). PARs are characterized by an activation mechanism whereby proteolytic cleavage at specific sites within the extracellular amino-terminus leads to the exposure of an amino-terminal “tethered ligand” domain. This new amino terminus is then able to affect transmembrane signaling (Vu et al., 1991). Thrombin is able to cleave PAR-1,-3, and -4 but not PAR-2 (Cunningham et al., 2000). TF, factor Xa, factor VIIa, trypsin, and mast cell tryptase, amongs others, are able to signal through PAR-2 (Camerer et al., 2000). While there is a wealth of data exploring the role of thrombin as an inflammatory mediator, there is yet to be a robust description of how thrombin acts on innate immune cells. This prompted us to investigate how thrombin signaling in monocyte/macrophages impacts the DTH response. We show that thrombin signaling through PAR-1 signaling plays a hitherto unrecognized and critical role in DTH responses, inducing downregulation of cell membrane reverse cholesterol transporter ATP-binding cassette transporter 1 (ABCA1) and increased expression of IFNγ receptor. These two key phenotypic changes combined to make thrombin-primed bone-marrow-derived macrophages extremely sensitive to M1 polarization.

## Results

### Inhibition of thrombin on CD31 + myeloid cells inhibits DTH responses to oxazolone

**Figure 1:**
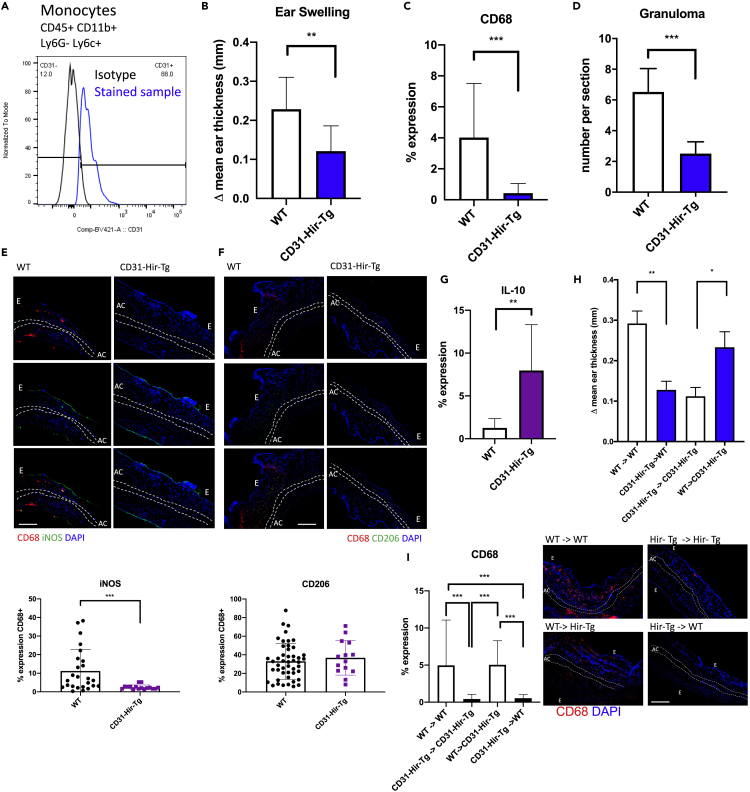
Results of oxazolone-induced delated-type hypersensitivity experiments in transgenic mice (A) Surface CD31 expression on monocytes (as defined as CD45 + CD11b + Ly6G- Ly6c+) on the peripheral blood of CD31-Hir-Tg mice. (B) Difference in ES at 24 hr. This is derived by subtracting thickness of the right ear (oxazolone) from that of the left (control) ear. WT group n = 6, transgenic group n = 6. (C) Immunofluorescence (IF) analysis of CD68 expression with the ear. Expression calculated by % lesion area occupied by CD68 + cells. (D) Granuloma assessed as the average number per section at 100× magnification. A granuloma was defined as a collection of CD68+/CD3+ cells outpouching from the epidermis. (E–G) IF analysis of proportion of CD68 + cells expressing iNOS (E), CD206 (F), or IL-10 (G). Graphs show percentage of CD68 cells that co-stain with iNOS or CD206 or in the case of IL-10, the % lesional area occupied by IL-10 + cells. Representative images show the following: CD68, red; CD206 or iNOS, green; DAPI, blue. The scale bar shows 200 μm in distance. (H) Bone marrow chimeric mice underwent oxazolone-induced DTH: graph shows change in ES at 24 hr compared to vehicle control ear. WT recipients of WT bone marrow and CD31-Hir-Tg recipients of CD31-Hir-Tg bone marrow represent experimental controls. Group numbers WT (CD45.1)- > WT (CD45.1) n = 3, CD31-Hir-Tg - > WT (CD45.1) n = 6, CD31-Hir-Tg - > CD31-Hir-Tg n = 6, WT (CD45.1) - > CD31-Hir-Tg n = 6. (I) IF results of macrophage infiltration (CD68, red; DAPI, blue) within the ear of the bone marrow chimeric mice. Associated graph shows expression calculated by % lesion area taken up by CD68 + cells when corrected for background florescence. The scale bar shows 200 μm in distance. Data are represented as mean ± standard error of mean (SEM). ∗P ≤ 0.05, ∗∗P ≤ 0.01, ∗∗∗P ≤ 0.001, ∗∗∗∗P ≤ 0.0001. E = epidermis, AC = auricular cartilage.

In order to investigate the role of thrombin in DTH responses, we induced a DTH response in the ear skin in response to oxazolone in either C57BL/6 wild-type (WT) or CD31-Hir-Tg mice. CD31-Hir-Tg mice express a fusion protein containing the direct thrombin inhibitor hirudin on all CD31 + cells including all circulating monocytes (Figure 1A) (Chen et al., 2004a). CD31-Hir-Tg mice had significantly reduced ear swelling (ES) compared to WT at 24 (p = 0.0019) and 48 (p = 0.0024) hours after re-challenge with oxazolone (Figure 1B). Immunofluorescence analysis of the ear sections revealed a reduction in the total number of macrophages as assessed by reduced CD68 + expression within the ear lesion from 4.9% in WT to 0.5% in CD31-Hr-Tg (p < 0.001) (Figure 1C), a reduction in the number of granulomas per section (Figure 1D) and a shift in the phenotype of recruited cells to a more anti-inflammatory profile with significantly reduced ratio of iNOS:CD206 expression on CD68 + cells coupled with an increase in IL-10 expression (Figures 1E–1G).

As the transgenic fusion protein in CD31-Hir-Tg mice is expressed on all CD31 + cells, we generated bone marrow (BM) chimeras with WT (CD45.1) mice to isolate expression on either BM-derived elements (platelets and monocytes) (Chen et al., 2004a) or ECs alone. Cells expressing CD45.1 allele (WT) can be distinguished from cells expressing the CD45.2 allele (CD31-Hir-Tg), allowing the easy tracking of donor and host leukocytes. Engraftment at day 30 was >95%. CD45.1 mouse recipients of CD31-Hir-Tg BM had a similar ES phenotype to parental CD31-Hir-Tg mice (Figure 1H), whereas CD31-Hir-Tg recipients of CD45.1 BM had a WT phenotype. Similarly, CD68 expression within the ear was reduced in the CD45.1 recipients of transgenic (CD-31-Hir-Tg) BM in comparison to CD31-Hir-Tg recipients of CD45.1 BM (Figure 1I).

**Figure 2:**
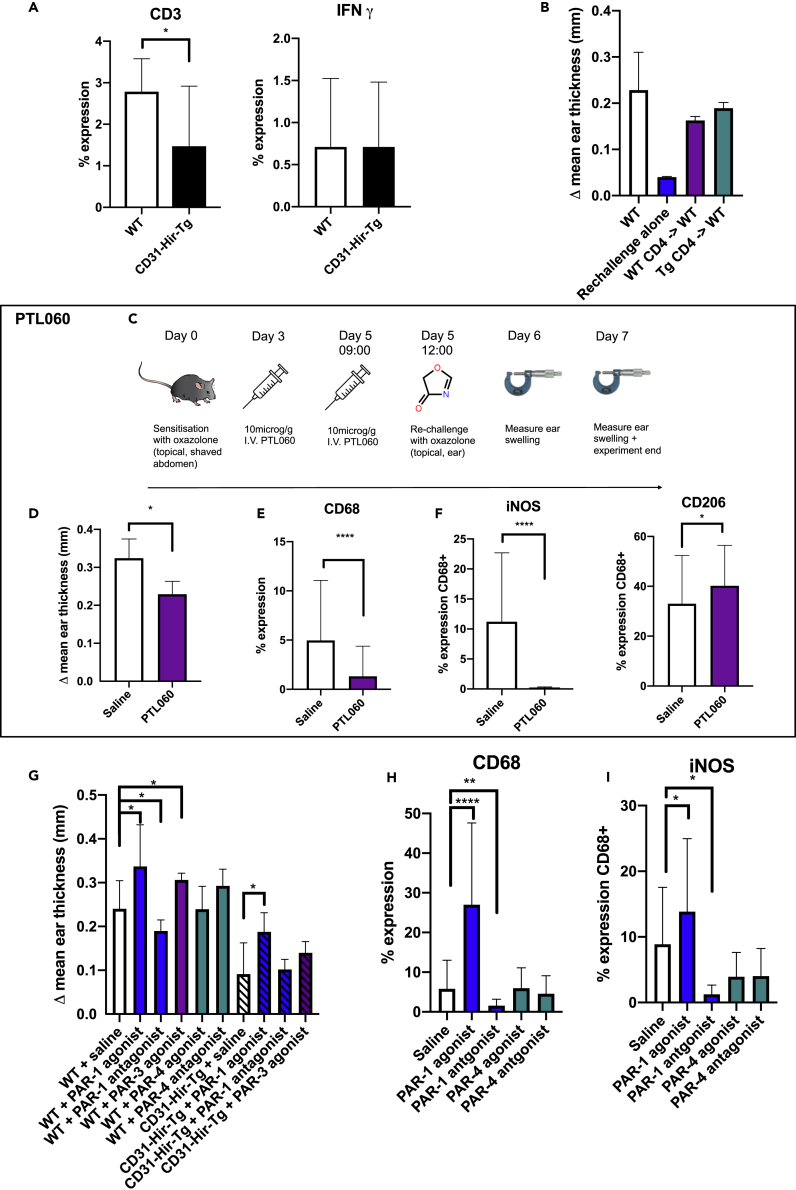
Assessing how transgenic expression of hirudin influences T-cell priming in type IV hypersensitivity and evaluating role of PAR signaling (A) CD3 and IFNγ expression in the WT or CD31-Hir-Tg mice after DTH. CD3 or IFNγ expression calculated by % lesion area taken up by CD3+ or IFNγ cells when corrected for background. WT group n = 6, transgenic group n = 6. (B) Adoptive transfer of oxazolone primed WT or CD31-Hir-Tg CD4 cells into WT mice before oxazolone applied to the ear. Change in ear thickness, compared to vehicle challenge alone, was measured at 24 hr. WT control mice received IV saline instead of cells but abdominal oxazolone challenge on day 0 and then ear re-challenge on day 5. “Re-challenge alone” mice were oxazolone-naive mice that received only 1% oxazolone in acetone and olive oil to the right ear. WT group n = 6, re-challenge alone n = 2, WT CD4 - > WT n = 4, Tg CD4 - > WT n = 6. (C) PTL060 experimental protocol. (D) ES results of WT (C57BL/6) mice treated with IV 10mcg/g PTL060 (n = 6) or equivalent volume saline (n = 4) on day 3 and 5 after sensitization in oxazolone-induced DTH model. (E) IF analysis of CD68 expression within the ear of PTL060-treated group vs saline. (F) IF analysis of iNOS and CD206 expression on CD68 + cells in the PTL060-treated group vs saline. (G) The effect of PAR signaling on DTH responses. Before a re-challenge on day 5, WT or CD31-Hir-tg mice received 10 microM/g intraperitoneal (IP) PAR-1 agonist (TFLLR-NH2) (n = 5) or antagonist (RWJ 56110) (n = 5) or PAR-4 agonist (GYPGQV trifluoroacetate salt) (n = 5) or antagonist (tcY-NH2) (n = 4) or PAR-3 agonist (H-Ser-Phe-Asn-Gly-Gly-Pro-NH2) (n = 5). The ears were then painted with oxazolone or vehicle alone. Data represent change in ES at 24 hr compared to the control ear. (H) IF analysis of CD68 expression within WT ears. Expression calculated by % lesion area taken up by CD68 + cells when corrected for background. (I) IF analysis of iNOS expression on CD68 + cells in WT ears. Data are represented as mean ± standard error of mean (SEM). ∗P ≤ 0.05, ∗∗P ≤ 0.01, ∗∗∗P ≤ 0.001, ∗∗∗∗P ≤ 0.0001.

There was a reduced T-cell (CD3+) infiltration into the ears of the CD31-Hir-Tg mice but no difference in IFNγ expression within the lesion (Figure 2A). To assess whether the expression of the transgenic fusion protein influenced T-cell priming, CD4+ T cells were isolated from the spleens of CD31-Hir-Tg or WT mice 5 days after initial exposure to oxazolone. These sensitized CD4+ T cells were then injected via the tail vein into oxazolone-naive WT mice who then underwent the usual re-challenge step with oxazolone. Recipients of CD31-Hir-Tg CD4 T cells had similar degrees of ES as recipients of WT controls (Figure 2B), indicating that CD4+ T-cell priming in CD31-Hir-Tg mice was “normal” and suggesting that the protective effect of the transgenic fusion protein was due to its expression on monocytes. There was no difference in circulating coagulation parameters: D-dimers, fibrinogen, thrombin antithrombin complex, TF, or thrombin activity between the WT and CD31-Hir-Tg mice (Figure S1), suggesting there was no systemic activation of coagulation proteases nor consumption of fibrinogen. However, the inflammation in control ears was accompanied by widespread local fibrin deposits, which were significantly diminished and appeared to be located predominantly only subepithelial in the oxazolone-treated ears of CD31-Hir-Tg mice, suggesting that the DTH response did involve local activation of coagulation proteases (Figure S2).

PTL060 is a cytotopic thrombin inhibitor based on Hirulog. On IV injection, a mirostyl tail anchors it into the lipid bilayer of circulating monocytes (and other cells) (Chen et al., 2020). When C57BL/6 mice undergoing DTH were treated with 10μg/g IV PTL060 on day 3 and day 5 (3 hr before re-challenge) (Figure 2C), there was a reduction in ES compared to saline control (p = 0.0121) (Figure 2D). Examination of the ears by immunohistochemistry revealed, in comparison to saline controls, PTL060 lead to a reduction in CD68 infiltration from 4.9% to 1.3% (p < 0.0001) (Figure 2E) and the adoption of a more anti-inflammatory profile with an increase in CD68 + cells expressing CD206 33%–40% (p = 0.0332) and completely inhibited iNOS expression on CD68 + cells (11%–0% < 0.0001) (Figure 2F).

We postulated that the transgenic fusion protein was most likely influencing the phenotype by blocking thrombin activation of PAR-1. Therefore, prior to re-challenge, mice were treated with intraperitoneal PAR-1 agonists or antagonists. WT mice treated with a PAR-1 agonist (TFLLR-NH2) had an increase in ES (p = 0.0279), CD68 expression (p=<0.0001) with increased iNOS expression (p = 0.0212) when compared to saline controls (Figures 2G, 2H, and 2I), whereas those treated with a PAR-1 antagonist (RWJ 56110) had reduced ES (p = 0.0322), CD68 expression (0.0036), iNOS expression (p = 0.0104) compared to saline controls (Figure 2G, 2H, and 2I). (Experiments using different PAR-1 agonists and antagonists yielded entirely consistent results [data not shown].) Treatment with PAR-4 agonist (GYPGQV trifluoroacetate salt) or antagonist (tcY-NH2) had no impact on the outcome of DTH. Although a PAR-3 agonist (H-Ser-Phe-Asn-Gly-Gly-Pro-NH2) increased ES in WT mice (Figure 2G), it did not significantly increase ES in CD31-Hir-Tg mice, whereas those treated with a PAR-1 agonist developed significantly increased ES (p = 0.0219) (Figure 2G), suggesting that only the provision of a PAR-1 signal on CD31-Hir-Tg cells was sufficient to overcome the effect of thrombin inhibition.

All these data suggest that local generation of thrombin at the site of antigen re-challenge leads to activation of PAR-1 that critically contributes to the development of the recall response; inhibition of thrombin on monocytes/macrophages either through transgenic expression of hirudin or local tethering of hirulog significantly inhibits the DTH.

### Macrophage responses to thrombin

**Figure 3:**
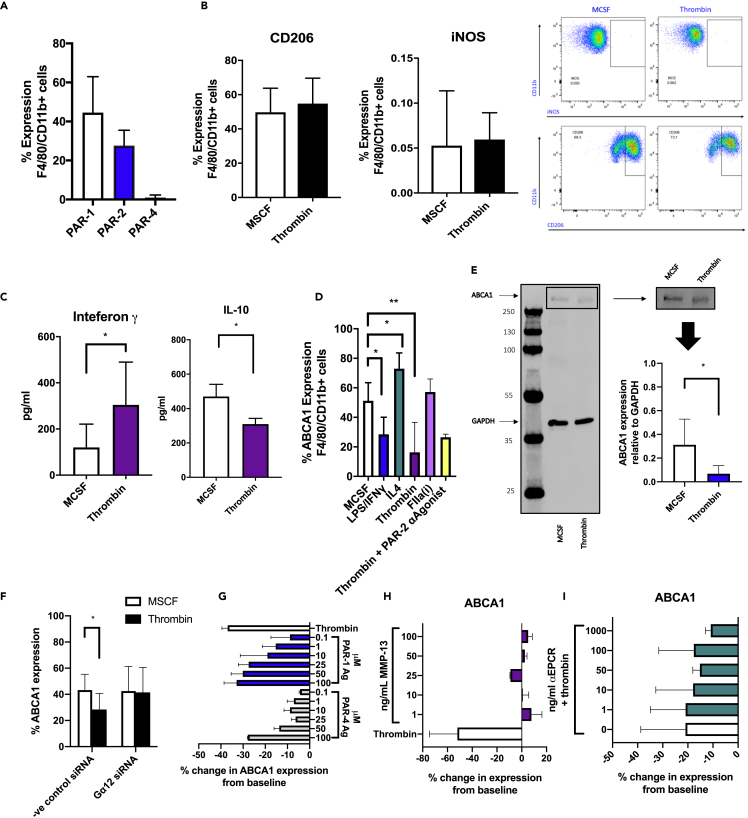
Thrombin induces a proinflammatory state without fully polarizing the cultured macrophages and downregulates ABCA1 expression through PAR-1 (A) Surface expression of PAR-1,-2, and -4 on bone marrow macrophages cultured for 5 days in complete bone marrow medium analyzed by flow cytometry. (B) Intracellular flow cytometric analysis of CD206 or iNOS expression on bone marrow macrophages cultured for 5 days in complete bone marrow medium and stimulated for 24 hr with 25 ng/ml MCSF or 50 units/ml of thrombin. Representative flow cytometry profiles are shown to the right. (C) Cell culture supernatants taken from cells treated for 24 hr with 25 ng/ml MCSF or 50 units/ml thrombin were analyzed by ELISA. IFNγ ELISA n = 5, IL-10 ELISA n = 4. ∗P ≤ 0.05. (D) ABCA1 expression, analyzed by flow cytometry, on F4/80 CD11b-positive cells after 5 days in bone marrow culture followed by 24 hr stimulation with 25 ng/ml MCSF, 100 ng/ml LPS, and 50 ng/ml IFNγ for M1 cells and 25 ng/ml IL4 for M2 cells or 50 units/ml thrombin or equimolar active site inhibited thrombin (FIIai) or the PAR-2 antagonist (PAR-2 αagonist) FSLLRY-NH2 for 2 hr prior to thrombin stimulation. Data are taken from at least 4 experiments. (E) Western blot of MCSF or thrombin-treated cells. Representative gel is shown to the right. ABCA1 band confirmed at approx. 250 kDA. (F) Surface ABCA1 expression of cells transfected with siRNA to Gα12 or negative control siRNA for 24 hr and then thrombin or MCSF for 24 hr. Data analyzed by flow cytometry. Data are taken from 3 experiments. (G) ABCA1 expression, analyzed by flow cytometry, on F4/80 CD11b-positive cells after 5 days in bone marrow culture followed by 24 hr stimulation with 25 ng/ml MCSF, thrombin, or increasing amounts of TFLLR-NH2 (PAR-1 agonist peptide) or GYPGQV trifluoroacetate salt (PAR-4 agonist peptide). Data represent percentage change in expression from control (MCSF) stimulated cells. (H and I) Change in surface ABCA1 expression analyzed by flow cytometry of bone marrow macrophages cultured for 24 hr with MMP-13 (H) or precultured for 2 hr with a neutralizing ePCR antibody prior to thrombin stimulation (I). Data shown as percentage change from baseline expression of MCSF media maintained cells in 3 different experiments. Data are represented as mean ± standard error of mean (SEM). ∗P ≤ 0.05, ∗∗P ≤ 0.01, ∗∗∗P ≤ 0.001, ∗∗∗∗P ≤ 0.0001.

To assess how thrombin signaling influences the behavior of WT macrophages, BM isolates were incubated with 25 ng/ml macrophage colony-stimulating factor (MCSF) for 5 days, which was found to be the time at which PAR-1 expression was maximal (Figure 3A). Cells were then stimulated for a further 24 hr with either thrombin or maintained in MCSF alone as a control. There was no change in iNOS or CD206 expression compared to baseline in response to thrombin (Figure 3B). Enzyme-linked immunosorbent assay (ELISA) confirmed a significant increase in IFNγ concentration in cell culture supernatants from thrombin-stimulated cells compared to controls (304.6 pg/mL vs 119.9pg/ml, respectively p = 0.0185), as well as a significant reduction in IL-10 production (454pg/ml vs. 309pg/ml p = 0.0286) (Figure 3C).

ABCA1 plays a critical role in lipid homeostasis and orchestrates the principal cellular pathway leading to cholesterol efflux (Pradel et al., 2009). We found that ABCA1 expression was highest in MCSF-matured BM cells that were treated with IL-4 for 24 hr and lowest after culture for 24 hr with a combination of LPS and IFNγ (Figure 3D). This is in keeping with previously published data (Singaraja et al., 2002). Next, we evaluated what role thrombin had on ABCA1 expression. Thrombin, but not active site-inhibited thrombin, down-regulated surface ABCA1 expression by flow cytometric analysis from 51.27% to 16.28% after 24 hr culture (p = 0.0024) (Figure 3D). Thrombin-mediated reduction in ABCA1 was also seen on Western blot (p = 0.0303) (Figure 3E). This was shown to be reliant on the G protein subunit Gα 12 as inhibiting this with small interfering RNA (siRNA) prevented thrombin-mediated ABCA1 downregulation (Figure 3F). Thrombin-cleaved PAR-1 is known to transactivate PAR-2 (O'Brien et al., 2000). Blocking the PAR-2 signal with the PAR-2 antagonist FSLLRY-NH2 prior to thrombin stimulation did not affect the outcome of thrombin on ABCA1 expression (Figure 3D). This thrombin-mediated ABCA1 downregulation was mimicked by culturing cells with the PAR-1 agonist peptide (TFLLR-NH2) (Figure 3G) and inhibited by antagonizing signaling through PAR-1 (Figure S3). Only at very high dose did PAR-4 agonist peptide (GYPGQV trifluoroacetate salt) impact ABCA1 expression (Figure 3G), whereas a PAR-3 agonist (H-Ser-Phe-Asn-Gly-Gly-Pro-NH2) failed to influence ABCA1 expression (Figure S4). Delivery of a signal through matrix metalloproteinase (MMP) 13 did not affect ABCA1 expression (Figure 3H). Noncanonical PAR-1 signaling can occur through the endothelial protein C receptor (ePCR) (Zhao et al., 2014). Preculturing the cells with an ePCR neutralizing antibody did not affect thrombin's ability to reduce ABCA1 expression (Figure 3I).

**Figure 4:**
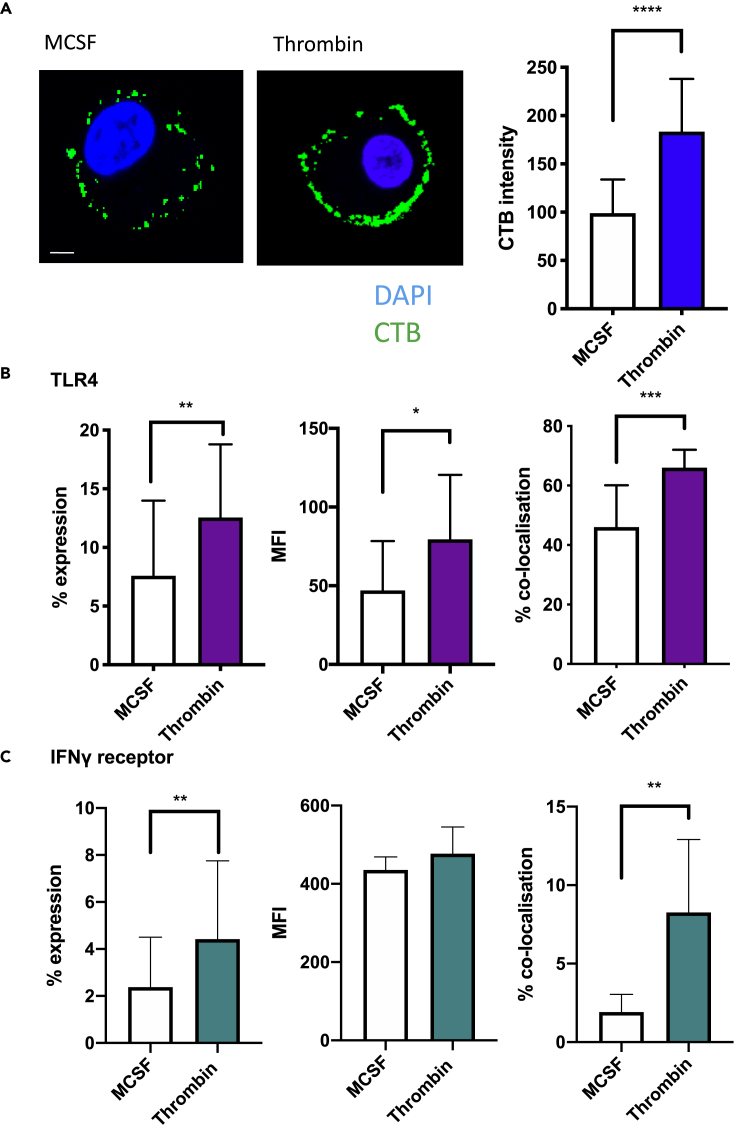
Thrombin increases the lipid raft content of cells (A) CTB (green) or DAPI (blue) staining of MCSF and thrombin-treated cells. The scale bar shows 10 μm in distance. Associated graph shows graphical representation of CTB intensity. (B) TLR4 surface expression as measured by IF on MCSF or thrombin-treated cells. Cells were prepared for lipid raft CTB staining as above and co-stained with fluorochrome-conjugated anti-TLR4 ab. The graphs represent, from left to right, the % of cells in positive gate, MFI of TLR4 on cells in the positive gate, and proportion of cells showing co-localization of CTB with TLR4. Costaining calculated using ICY cell imaging software using Pearson correlation coefficient of both CTB staining and TLR4 receptor staining. (C) IFNγ surface expression as measured by IF on MCSF or thrombin-treated cells. Cells were prepared for lipid raft CTB staining as above and co-stained with fluorochrome-conjugated anti-IFNγ ab. The graphs represent, from left to right, the % of cells in positive gate, MFI of IFNγ on cells in the positive gate, and proportion of cells showing co-localization of CTB with IFNγ. Costaining calculated using ICY cell imaging software using Pearson correlation coefficient of both CTB staining and IFNγ receptor staining. Data are shown from 4 separate experiments. Data are represented as mean **±** standard error of mean (SEM). ∗P ≤ 0.05, ∗∗P ≤ 0.01, ∗∗∗P ≤ 0.001, ∗∗∗∗P ≤ 0.0001.

ABCA1 has been linked to the formation of lipid-rich microdomains in the external leaflet of the plasma membrane (Zhu et al., 2010). These discrete lipid domains, representing organized accumulations of cholesterol and glycosphingolipids, play a key role in inflammatory signaling due to the high concentration of cell receptors residing within the “lipid rafts” (Pike, 2003). To evaluate the role thrombin signaling had on lipid rafts, bone marrow derived macrophages (BMM) were incubated for 24 hr in complete media with MCSF or thrombin. After 24 hr, cells were stained using Vybrant Alexa Fluor 488 Lipid Raft Labeling Kit. The thrombin-treated cells had increased expression of cholera toxin B (CTB) on the cell surface, correlating with increased lipid raft formation (p < 0.0001) (Figure 4A). Surface expression of TLR4 increased upon thrombin stimulation (mean fluorescence intensity [MFI] increased from 47.01 to 79.02 [p = 0.0427]), and there was also increased colocalization of the receptor within the lipid rafts (46.04% vs. 66.03% p = 0.0004) (Figure 4B). Thrombin stimulation increased surface expression of IFNγ receptor (MFI 435.6 vs 477.4) (p = 0.0287), and these also showed increased expression within the lipid rafts from 2.39% expression to 8.73% (p = 0.0031) (Figure 4C).

### Thrombin primes BM-derived macrophages to be hyperresponsive to M1 polarizing signals

**Figure 5:**
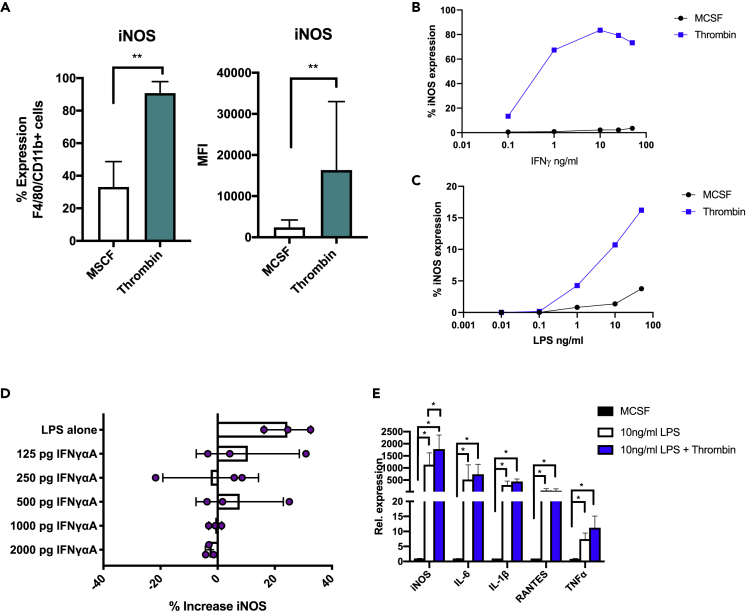
Priming with thrombin increases sensitivity to low-dose LPS and IFNγ (A) Intracellular flow cytometric analysis of % of iNOS + cells and MFI of iNOS expression by cells in the positive gate. Cells were murine bone marrow macrophages primed for 24 hr with thrombin or control (MCSF) n = 6 prior to 24-hr stimulation with low-dose M1 stimuli 0.01 ng/mL LPS and 50 ng/mL IFNγ n = 6. (B and C) BMMs were primed for 24 hr with MCSF or 50 units/ml thrombin as indicated and then stimulated for a further 24 hr with escalating amounts of either IFNγ alone (B) or LPS alone (C). Cells were then analyzed by intracellular flow cytometry for iNOS expression. Data represent % positive cells. (D) The effect of IFNγ blockade on heightened sensitivity to LPS alone. BMMs were cultured for 5 days with 25 ng/ml MCSF and then stimulated for 24 hr with thrombin. Media was replaced with fresh media containing escalating doses of IFNγ blocker (IFNγαag) (Abcam) for 1 hr. All wells were then treated with low-dose LPS (10 ng/ml) +/− thrombin for 24 hr. iNOS expression was then analyzed by flow cytometry. Data shown change in iNOS expression between control and thrombin-treated cells. Each data point represents a single experiment; bars represent mean of data. (E) qPCR data for the expression of TNFα, IL-1β, IL-6, RANTES, and iNOS. BMMs were stimulated for 24 hr with thrombin or maintained in complete media. After 24 hr, the media was removed and replaced with fresh media containing 10 ng/ml LPS +/− thrombin. Cells were removed for qPCR analysis 4 hr later. Data shown relative to MCSF control cells. Data are shown from 4 separate experiments. Error bars are means of data. Data are represented as mean ± standard error of mean (SEM). ∗P ≤ 0.05, ∗∗P ≤ 0.01, ∗∗∗P ≤ 0.001, ∗∗∗∗P ≤ 0.0001.

Given thrombin's apparent role in augmentation of lipid raft composition, specifically with the increase in both the LPS and IFNγ receptor—both moderators of the M1 phenotype—we considered that thrombin was priming the cells which could potentially translate to increased responsiveness to LPS or IFNγ. For these experiments, BMM were incubated for 24 hr with thrombin or MCSF alone as a control followed by increasing concentrations of LPS and/or IFNγ. Thrombin-stimulated cells were more sensitive to the combination of low dose LPS/IFNγ, evidenced by increased proportion of iNOS expression (29.1% vs 89.3% p = 0.0079) and increased MFI (1543 vs. 9096 p = 0.0040) (Figure 5A). Thrombin-stimulated cells were exquisitely sensitive to very low dose IFNγ (in the absence of LPS) with increasing concentrations resulting in enhanced iNOS expression in a dose-dependent manner (Figure 5B). Similarly, but to a lesser extent, the cells were also sensitive to low-dose LPS (without IFNγ) (Figure 5C). These enhanced responses to low-dose LPS appeared to be due entirely to thrombin-mediated increases in IFNγ secretion, as they were abolished by increasing amounts of an IFNγ blocking antibody (Figure 5D).

LPS stimulation of the BMM increased the expression of iNOS (p = 0.0286), TNFα (p = 0.0030), RANTES (p = 0.0079), IL-6 (p = 0.0286), and IL-1β (p = 0.0287) by qPCR. Pre-treatment with thrombin increased further iNOS expression (p = 0.0286) during LPS stimulation, but this heightened sensitivity to LPS was not seen in TNFα, RANTES, IL-6, or IL-1β expression (Figure 5E). LPS and PAR-2 have been shown to synergistically enhance inflammatory signaling (Ostrowska et al., 2007). There was no difference in PAR-2 expression during thrombin stimulation, so the enhanced responses to low-dose LPS cannot be attributed to increased PAR-2 expression (Figure S5).

**Figure 6:**
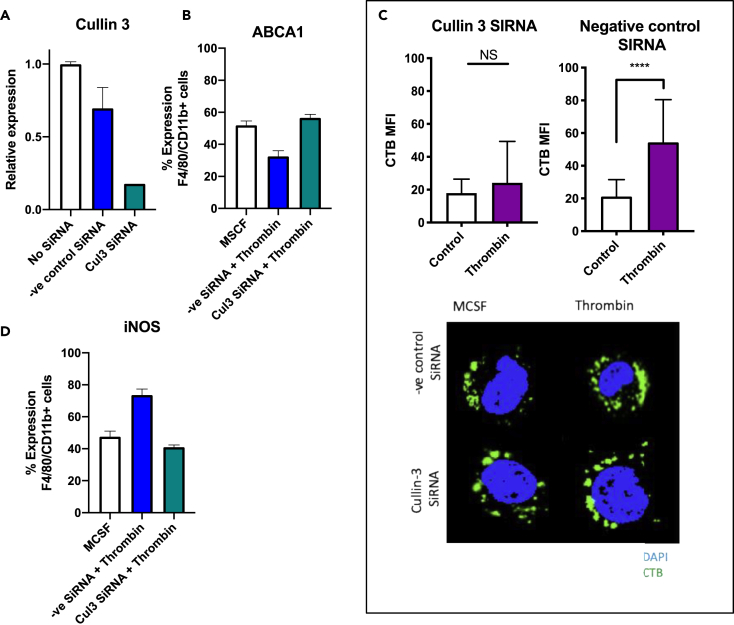
ABCA1 is essential for heightened sensitivity of thrombin-primed cells to LPS and IFNγ (A) Cullin 3 expression, analyzed by qPCR, after 24 hr transfection of BMM cultured for 24 hr with cullin 3 siRNA, negative control siRNA, or control cells maintained in complete media containing 25 ng/ml MCSF. (B) The above cells were then stimulated for 24 hr with thrombin, and surface ABCA1 expression was assessed by flow cytometry. (C) CTB staining of lipid rafts of the three experimental cell groups after 24 hr siRNA (or control) transfection and then 24 hr of thrombin. Cells were counterstained with DAPI, then analyzed using an inverted confocal microscope at 60× magnification (oil immersion), and analyzed using NIS-Elements software. The scale bar shows 10 μm in distance. (D) The cells were then treated for a further 4 hr with 0.01 ng/ml LPS and 50 ng/ml IFNγ with or without thrombin and then analyzed by flow cytometry for intracellular iNOS expression. Data are shown from 3 different experiments. Data are represented as mean ± standard error of mean (SEM). ∗P ≤ 0.05, ∗∗P ≤ 0.01, ∗∗∗P ≤ 0.001, ∗∗∗∗P ≤ 0.0001.

Thrombin-mediated downregulation of ABCA1 has been described to be via upregulation of the ubiquitin-proteasome system component cullin 3 (Raghavan et al., 2018). To assess the importance of ABCA1 to thrombin-mediated heightened sensitivity to low-dose M1 stimuli, cullin 3 siRNA was used to maintain ABCA1 expression (Figures 6A, 6B, and S6) in the face of thrombin stimulation. This inhibition of thrombin-mediated ABCA1 downregulation by cullin 3 siRNA was associated with a failure to increase cell membrane lipid rafts (Figure 6C) and a loss of the hypersensitivity to low-dose LPS/IFNγ seen after exposure to thrombin (Figure 6D).

Taken together, all these data indicate that thrombin, through PAR-1 signaling, primes BMM to polarization by IFNγ and TLR4 agonists. This is via an increase in expression of IFNγ, IFNγ receptor and TLR4, and co-localization of both receptors in membrane lipid-rich microdomains, due to the associated downregulation of ABCA1 by cullin 3.

### ABCA1 is critical to the phenotype of ES in delayed-type hypersensitivity

**Figure 7:**
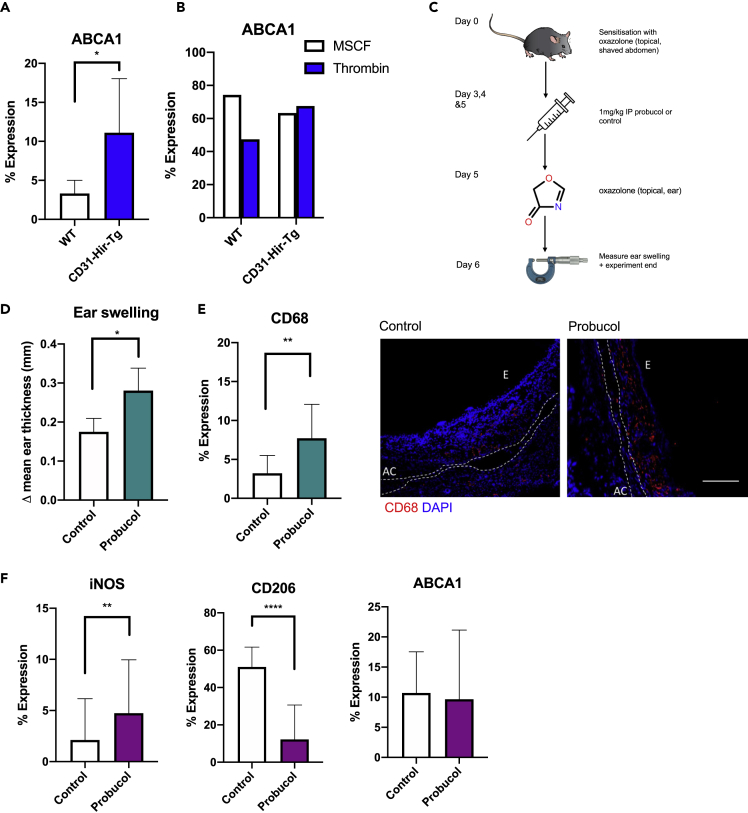
The importance of ABCA1 *in vivo* (A) ABCA1 expression in the ears of WT or CD31-Hir-Tg mice after oxazolone-induced DTH measured by IHC. Represented as % of CD68 + cells co-expressing ABCA1. WT group n = 6, transgenic group n = 6. (B) Flow cytometric analysis of surface ABCA1 expression of cultured WT or CD31-Hir-Tg BMM treated with 24 hr 50U/ml thrombin or MCSF control. (C) IP probucol experiments. CD31-Hir-Tg mice were challenged with 5% oxazolone on day 0. Then, from day 2–5, they received 1 mg/kg IP probucol (n = 4) or control (n = 4) before re-challenge with oxazolone on day 5. (D) Data represent difference in ES at 24 hr (E) IP probucol experiments. IF analysis of CD68 expression with the ear. Expression calculated by % lesion area taken up by CD68 + cells when corrected for background. Representative images show the following: CD68, red; DAPI, blue. E = epidermis, AC = auricular cartilage. The scale bar shows 200 μm in distance. (F) IP probucol experiments. IF analysis of CD206, iNOS, and ABCA1 expression on CD68 + cells. Data are represented as mean ± standard error of mean (SEM). ∗P ≤ 0.05, ∗∗P ≤ 0.01, ∗∗∗P ≤ 0.001, ∗∗∗∗P ≤ 0.0001.

To confirm that these mechanistic steps were operational in the DTH responses *in vivo*, we confirmed that CD31-Hir-Tg mice showed increased ABCA1 expression compared to WT mice (Figure 7A) after second exposure to oxazolone. BM isolates from CD31-Hir-Tg were not sensitive to thrombin and thus maintained ABCA1 expression in the face of thrombin (Figure 7B). Finally, CD31-Hir-Tg mice were treated with IP probucol for 3 days prior to oxazolone re-challenge (Figure 7C). Probucol inhibits ABCA1-mediated cellular lipid efflux but does not affect ABCA1 surface expression (Favari et al., 2004). The probucol-treated CD31-Hir-Tg mice had an increase in ES at 24 and 48 hr compared to saline-treated control CD31-Hir-Tg mice (Figure 7D), associated with increased infiltration by CD68 + cells (Figure 7E), expressing reduced levels of CD206 but increased levels of iNOS (without any change in ABCA1 expression) (Figure 7F).

## Discussion

In this study, we describe how the serine protease thrombin is able to prime macrophages to become exquisitely responsive to low doses of LPS and IFNγ. We confirm the reports of others (Chinetti-Gbaguidi et al., 2015) that ABCA1 is a marker of IL-4 stimulated anti-inflammatory macrophages. Moreover, we describe the link between thrombin stimulation, lipid raft composition alteration, and increased sensitivity to M1 stimuli. Finally, to our knowledge, we provide the first report of ABCA1's key role in the development of normal DTH responses and the first report that thrombin-mediated PAR-1 signaling provides the stimulus *in vivo* for ABCA1 downregulation.

Thrombin is the main effector protein in the coagulation cascade (Manabe et al., 2009) but is able to directly affect a wide array of cell types such as smooth muscle cells, platelets, and ECs (Cunningham et al., 2000) via signaling through PARs. We have previously described the roles that thrombin plays in acute and chronic vascular inflammation using CD31-Hir-Tg mice (Chen et al., 2006, 2008a, 2008b). In a mouse-to-rat model of heart transplantation, hearts from CD31-Hir-Tg mice rejected significantly later compared to WT hearts (Chen et al., 2004b), due to inhibition of both intravascular thrombosis associated with antibody-mediated rejection (AMR) and inhibition of thrombin-dependent CCL2 chemokine gradients necessary for monocyte recruitment (Chen et al., 2006, 2008b) in this model. Aortas from these mice, when transplanted into apolipoprotein E (ApoE)−/− mice fed a high-fat diet, fail to express CCL2 and MIF and do not develop atherosclerosis, in contrast to the florid lesions seen in control WT aortas (Chen et al., 2020). Recently, we have reported pretransplant perfusion into rat or primate organs with PTL060 (or related compounds) prevents the intravascular thrombosis associated with AMR (Manook et al., 2017; Karegli et al., 2017). Most recently, we have showed that intravenous delivery of PTL060 into ApoE−/− mice fed a high-fat diet leads to widespread coating of the endothelium, inhibits expression of both CCL2 and MIF, and prevents atheroma formation (Chen et al., 2020). Importantly, in this work, intravenous delivery of PTL060 also led to widespread uptake onto the membranes of circulating leukocytes and was associated with significant regression of atherosclerotic plaques when treatment was started 16 weeks after the beginning of the high-fat diet (Chen et al., 2020). In this model, the direct effect of PTL060 on monocytes was the dominant mechanism driving atheroma regression, as the same phenotype was achieved by adoptive transfer of PTL060-coated monocytes.

The data in a contact dermatitis model, presented here, are entirely consistent with our data in atherosclerosis but provide a much greater mechanistic insight into the role and importance of thrombin in monocyte/macrophage polarization *in vivo*. Expression of a hirudin fusion protein on monocytes prevented ES after second exposure to oxazolone and shifted the phenotype of DTH lesions away from an M1 spectrum toward M2. This was PAR-1 and ABCA1 dependent. Furthermore, we have demonstrated that PTL060 also delivers a protective phenotype in this additional model system.

Our *in vitro* experiments revealed the mechanistic basis of these findings. Thrombin, via PAR-1-mediated ABCA1 downregulation, increased the expression of IFNγR and shifted the receptors into cholesterol-rich microdomains, resulting in a massively increased sensitivity to IFNγ-mediated polarization. At the same time, TLR4 expression was increased within the same lipid rafts, and thrombin induced secretion of picomolar concentrations of IFNγ, which in combination, enhanced the sensitivity of cells to LPS-mediated polarization. Thrombin's nuanced role in LPS stimulation was further highlighted when pre-treatment with thrombin changed the expression of some (iNOS) but not all Myd88 and TRIF-dependent genes during TLR4 stimulation (Leifer and Medvedev, 2016). *In vitro* there appeared to be a correlation between high dose PAR-4 stimulation and ABCA1 expression. This is not entirely surprising as it is well documented that the PAR-4 receptor lacks the hirudin-like domain; therefore, higher concentrations of thrombin are required to initiate cellular signaling (Xu et al., 1998). We did not see any evidence of PAR-4 signaling affecting the outcome of the *in vivo* findings. In contrast, a PAR-3 agonist even at high doses failed to impact on ABCA1 expression, and although the same agent caused increase ES in WT mice, it did not partially reverse the phenotype of the CD31-Hir-Tg mice in the same way as a PAR-1 agonist, suggesting a minor role, if any for PAR-3 in this model. That said, a definitive conclusion about the role of PAR-3 is difficult in the absence of reliable reagents to antagonize PAR-3 activation.

We believe that, in this model, the thrombin is generated on the surface of myeloid cells, which are known to express TF (Rao and Pendurthi, 2012). We were able to demonstrate evidence of local fibrin generation in the WT mice but not the transgenic strain. Interestingly, there was no evidence of systemic activation of coagulation.

Other groups have previously reported on the impact of PAR-1 signaling on monocyte/macrophage function. In RAW cells, thrombin has been shown to induce iNOS (Kang et al., 2003). In human THP1 cells, thrombin has been linked to IL-8 production (Kang et al., 2003). In a model of *Citrobacter rodentium*-induced colitis, PAR-1 signaling on monocytes was shown to be key to promoting Th17-type immune response via IL-23 (Saeed et al., 2017). PAR-1 signaling has been shown to enhance the Poly I:C induction of the antiviral response via TLR3 in bone marrow macrophages (Antoniak et al., 2017). Recently, López-Zambrano et al. reported that thrombin signaling, in part through PAR-1, was sufficient to induce M1 polarization in bone marrow macrophages (Lopez-Zambrano et al., 2020). The difference between our data and this work is likely to be due to the use of L929 conditioned medium to differentiate the BMM instead of purified MCSF. Taken together, our data are consistent with the underlying implication that thrombin primes monocytes to make enhanced responses to microenvironmental polarization cues. Priming of monocytes has been described by others. Askenase et al. have recently described how monocytes are primed for regulatory function prior to egress from the bone marrow using a model of gastrointestinal infection. In this model, natural killer cell-derived IFNγ promoted regulatory programming in monocyte progenitors controlled by systemic IL-12 produced by Batf3-dependent dendritic cells in the mucosa-associated lymphoid tissue (Askenase et al., 2015). Our data suggest that the sensitivity of monocytes to distal priming by systemic cytokines may be regulated by cell-intrinsic mechanisms controlling the encryption and de-encryption of TF on myeloid cells, which is known to regulate their ability to generate cell surface thrombin and other coagulation proteases (Chen and Hogg, 2013).

ABCA1 is a major regulator of cellular cholesterol and phospholipid homeostasis (Singaraja et al., 2002). It has a key role in atherosclerosis, mediating the efflux of cholesterol and phospholipids and thus reducing the atherosclerotic plaque burden (Pradel et al., 2009). Our data are consistent with other reports that ABCA1 is linked to an anti-inflammatory M2 phenotype (Pradel et al., 2009) and augment the report from Raghavan et al., which first revealed that thrombin downregulates ABCA1 expression (Raghavan et al., 2018) via cullin 3 expression, which is a component of cullin-RING E3 ubiquitin ligase complex involved in protein ubiquitination (Dubiel et al., 2018). ABCA1 has been shown to disrupt cholesterol-rich microdomains via redistribution of cholesterol from rafts to non-rafts through its ATPase-related functions (Zhu et al., 2010). In our study, we were able to show a direct link between thrombin stimulation, ABCA1 down regulation, increase in lipid-rich microdomains at the cell membrane, and increased sensitivity to IFNγ, which, along with the secretion of picomolar concentrations of IFNγ, was the basis for the increased sensitivity to LPS. This is consistent with previous reports that human monocytes when cultured with IFNγ have heightened responses to bacterial LPS (Hayes and Zoon, 1993).

In summary, we have provided the first evidence that thrombin mediated PAR-1 signaling on the surface of monocytes, leading to ABCA1 downregulation and an associated sensitivity to IFNγ, and TLR stimulation is critically involved in the development of normal DTH responses *in vivo*. Targeting this pathway could potentially offer a way to modulate innate immune responsiveness and to control inflammatory responses in multiple diseases.

### Limitations of the study

A potential limitation of this work is that we have not confirmed results in mice-deficient in PAR, particularly PAR-1. Our rationale is that both the priming/sensitization and re-challenge phases would be influenced by the lack of PAR-1 signaling. Our approach instead relied upon using highly specific agonists and antagonists to allow us to isolate only the re-challenge phase for study.

We have also not addressed the role of PAR-2 signaling in this model, as our ongoing experiments dissecting the impact of PAR-2 stimulation suggest complex interactions between PAR-2 and PAR-1 stimulation which require further interrogation and will be the subject of a subsequent report. Others have reported that PAR-2 signaling in contact dermatitis contributes to the inflammatory response (Seeliger et al., 2003).

### Resource availability

#### Lead contact

Further information and requests for resources should be directed to and will be fulfilled by the Lead Contact, Hannah Wilkinson (hannah.wilkinson@kcl.ac.uk).

#### Material availability

This study did not generate new unique reagents.

#### Data and code availability

This study did not generate data sets or codes.

## Methods

All methods can be found in the accompanying Transparent methods supplemental file.

## Supplemental information

Document S1. Transparent methods and figures S1–S6
