# Supplementary material for: PAR-1 signaling on macrophages is required for effective in vivo delayed-type hypersensitivity responses
Source: iScience. 2021 Jan 5;24(1):101981. doi: 10.1016/j.isci.2020.101981 (PMC7797913; doi:10.1016/j.isci.2020.101981)
Supplement: Document S1. Transparent methods and figures S1–S6 [file mmc1.pdf]

iScience, Volume *24*

Supplemental Information

PAR-1 signaling on macrophages is required for effective in vivo delayed-type hypersensitivity responses

Hannah Wilkinson, Hugh Leonard, Daxin Chen, Toby Lawrence, Michael Robson, Pieter. Goossens, John H. McVey, and Anthony Dorling

Supplemental Figure 1.

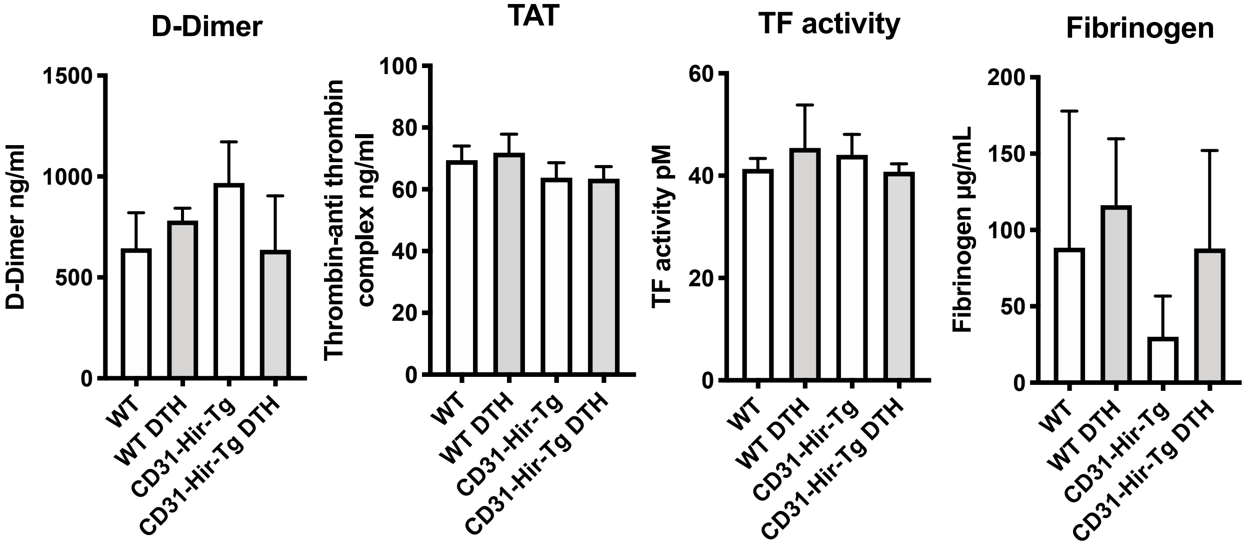

Supplemental Figure 1. Parameters of coagulation in Tg and WT mice. Related to Figure 1. Plasma TF activity was measured using an activity assay commercially available from Abcam. Plasma D-Dimer, fibrinogen and TAT complex were measured by ELISA in CD31-Hir-Tg and WT mice at baseline and after delayed type hypersensitivity (designated WT DTH or CD31-Hir-Tg DTH). Data are represented as mean ± SEM.

Supplemental Figure 2.

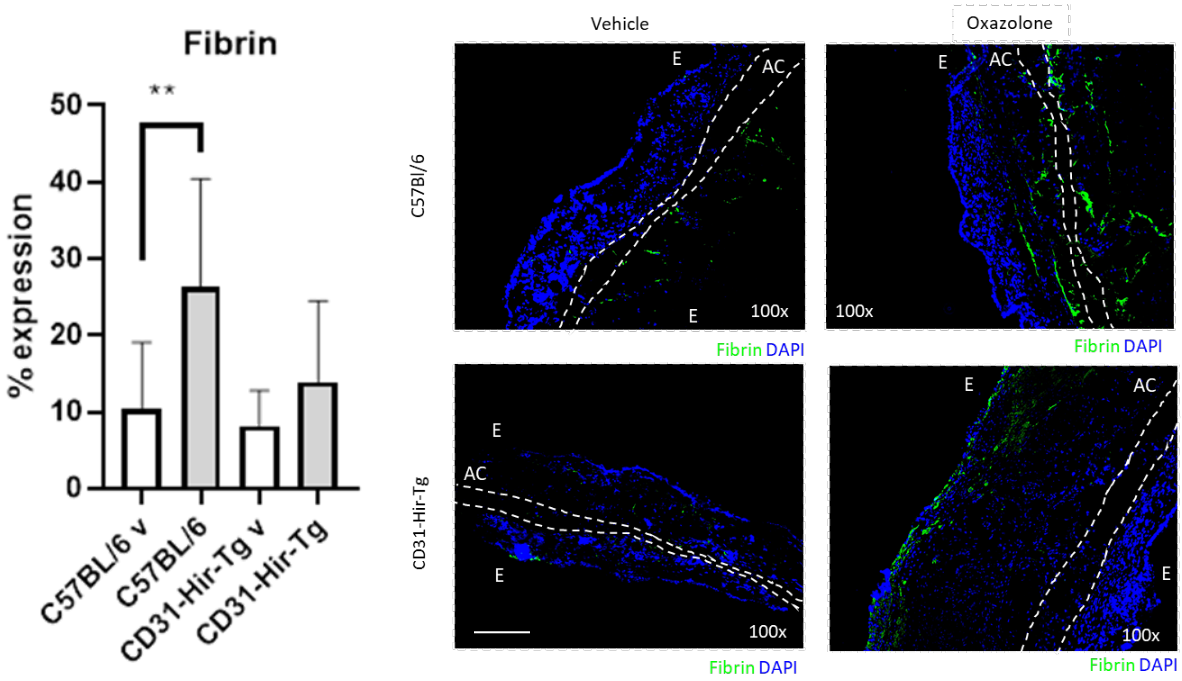

Supplemental figure 2. Fibrin expression in the ears of WT or CD31-Hir-Tg mice after DTH. Related to Figure 1.

Immunofluorescence analysis with associated graphical representation, of Fibrin in the oxazolone and vehicle (v) treated ears of CD31-Hir-Tg and WT mice. Expression calculated by % ear area occupied by Fibrin. Images are representative and show Fibrin (green) with DAPI (blue) E= epidermis, AC= auricular cartilage. Data are represented as mean ± SEM. The scale bar shows 200 μm in distance.

Supplemental Figure 3.

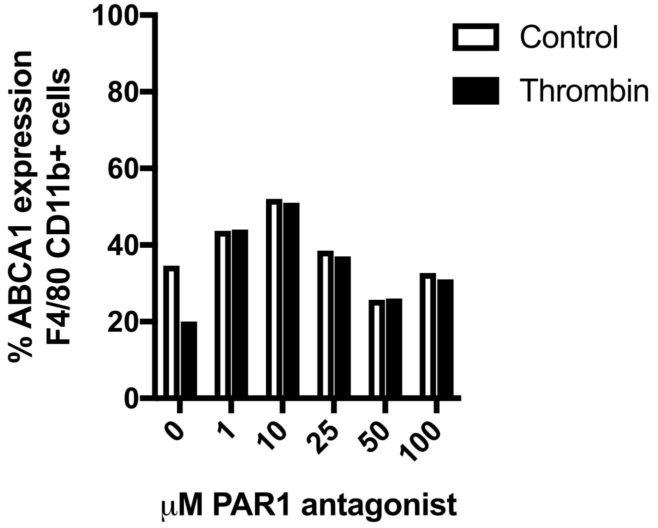

Supplemental Figure 3. ABCA1 expression in response to PAR-1 antagonists. Related to Figure 3G.

BMM were incubated with increasing amounts of PAR-1 antagonist (FLLRN) for 1 hour prior to the addition of thrombin for a further 23 hours. Control cells were maintained in the PAR-1 antagonist throughout the 24 hours assay. Cells were then analysed for surface ABCA1 expression by flow cytometry. Data are represented as mean ± SEM.

Supplemental Figure 4.

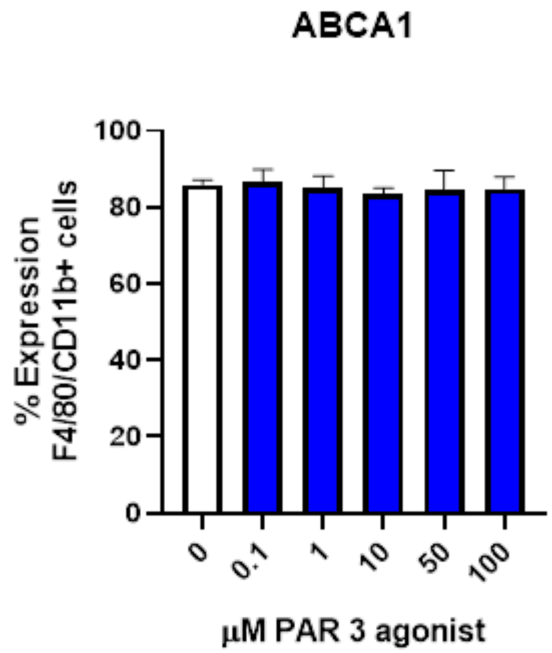

Supplemental Figure 4. ABCA1 expression in response to PAR-1 antagonists. Related to Figure 3G. ABCA1 expression, analysed by flow cytometry, on F4/80 CD11b positive cells after 5 days in bone marrow culture followed by 24 hours stimulation with 25ng/ml MCSF, thrombin, or increasing amounts of H-Ser-Phe-Asn-Gly-Gly-Pro-NH2 (PAR3 Tethered Ligand) Data are represented as mean ± SEM.

Supplemental Figure 5.

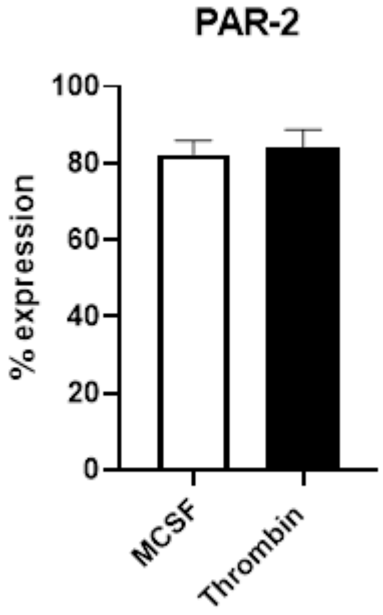

Supplemental Figure 5. PAR-2 expression in response to thrombin stimulation. Related to Figure 3.
Surface PAR-2 expression analysed by flow cytometry on BMM after 24 hours stimulation with 50 units/ml of thrombin or maintained in control media with MCSF. Data are represented as mean ± SEM.

Supplementary Figure 6.

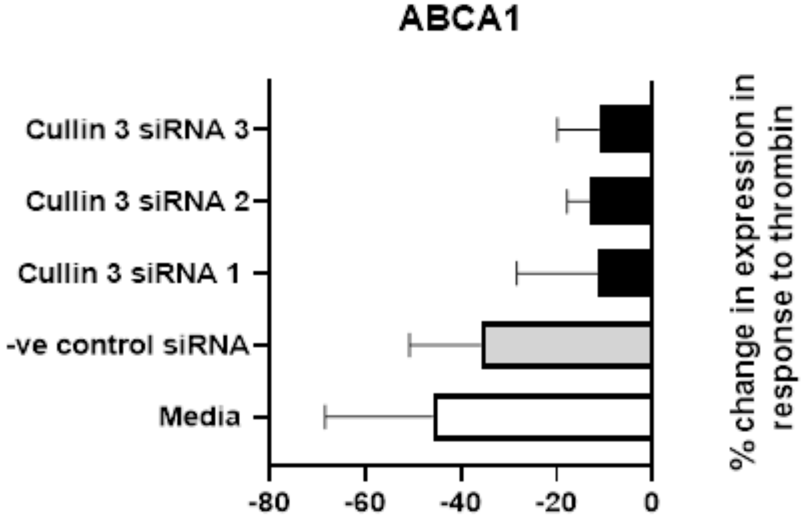

Supplementary Figure 6. Optimization of Cullin 3 siRNA. **Related to Figure 6.**
Three siRNAs to cullin 3 were purchased from Thermofischer scientific. (ID siRNA 1-188654; siRNA 2-72389; siRNA3-188655). Cells were transfected for 24 hours prior to thrombin stimulation. Cells were then analysed by flow cytometry for ABCA1 expression. Cullin 3 siRNA # 1 was used in the main data section.Data are represented as mean ± SEM.

Transparent Methods

Animals: 6-12 week old male C57BL/6 mice were purchased from Envigo and housed in specific pathogen free environment. CD45.1 and CD31-Hir-Tg (Chen et al., 2004) mice were bred in house on a C57BL/6 background. All procedures were performed in accordance with the Home Office Animals (Scientific Procedures) Act of 1986.

Delayed type hypersensitivity experiments: On Day 0 a 50μl preparation of 5% oxazolone (Sigma, Dorset, UK) in ethanol and acetone (4:1) was applied to the shaved abdomen. Mice were re-challenged on day 5 by applying 1% oxazolone in olive oil and acetone (4:1, 10 μl) to the right ear and vehicle alone to the left ear. Ear thickness was measured using a digital micrometre using at least 5 measurements and this was subtracted from the mean ear thickness of the vehicle treated ear After 48 hours the mice were sacrificed by schedule one methods and the ears removed and added to a cryomold and covered in OCT. Samples were stored at -80ºC prior to analysis. In vivo mice were treated with IP PAR agonists/antagonists at the molarity described in the experiments prior to rechallenge on day 5. For the PTL060 experiments mice received 10μg/g IV Thrombalexin on day 3 and day 5 (3 hours before re-challenge). For the probucol experiments CD31-Hir-Tg mice received 1mg/kg IP probucol (Stratech, Ely, UK) daily on days 2-5. Immediately after last IP injection, the mice were re-challenged with 1% oxazolone in olive oil and acetone (4:1, 10 μl) to the right ear and vehicle alone to the left ear.

T cell isolation and adoptive transfer: Mice were sensitised with oxazolone to the shaved abdomen as described above. After 5 days they were culled by a schedule 1 method and their spleens were removed and placed over a 70micron filter. The tissue was disrupted with a plunger and the filter was flushed with 15mls cold DMEM. Red cells were lysed by incubation with ammonium chloride lysis buffer and CD4 cells were isolated using CD4 MicroBeads (Miltenyi Biotech, Woking, UK) as per manufacturer's instruction. 5x10^6 purified CD4+ cells were then injected via the tail vein into naïve recipient WT mice, which were then challenged with 1% oxazolone in olive oil and ethanolto the right ear and vehicle alone to the left. ES was measured at 24 and 48 hours.

Bone marrow transplant: Recipient mice were irradiated with 9 Gy and then reconstituted with $5x10^6$ bone marrow cells (see below for isolation protocol) intravenously via tail vein within 24 hours of irradiation. Mice were weighed daily and monitored for signs of distress. Engraftment was assessed by surface CD45.1 and CD45.2 expression on peripheral blood cells acquired through tail vein venepuncture by flow cytometry after day 30.

Immunofluorescence analysis: Tissue sections were cut (5μm) using a cryostat (Bright Instrument Ltd, Huntington, UK) and transferred onto multispot glass slides (Hendley-Essex, Loughton, UK). Sections were fixed in methanol for 1 hour at -20ºC and then left to air dry. Sections were then blocked with 10% foetal calf serum (FCS) in PBS for 1 hour after which they were washed 3 times for 5 minutes in PBS 0.5% Triton X-100. Primary antibodies used in this study were rat anti mouse CD68 (FA-11 Thermofischer Scientific, UK) and CD3 (ab5690 Abcam, Cambridge, UK) and rabbit anti mouse CD206 (ab64693 Abcam), iNOS (ab15323 Abcam), ABCA1 (ab7360 Abcam), IL-10 (ab34843 Abcam) and IFNγ (ab9657 Abcam). Primary antibodies were incubated overnight in a humidified chamber, before washing and application of the secondary antibody (goat anti-rat AF594 or goat anti-rabbit AF488 (Abcam)) for 2 hours at room temperature (RT). Slides were mounted with Vectashield Antifade Mounting Media with DAPI (2BScientific, Oxford, UK) and covered with glass cover slips. All sections were stored in the dark at 4 ºC before analysis using a fluorescence microscope. For quantification, images were assessed at 100X magnification, background signal was assessed with isotype and no primary controls. Using ImageJ software, the area of the lesion was drawn around and percentage expression assessed using threshold measurements to remove background signal. At least 5 images were taken per section. Colocalisation analysis was performed using Pearson correlation analysis on ICY software.

Bone marrow isolation protocol: Bone marrow cell suspensions were isolated by flushing femurs and tibias of 8-12 week-old donor mice with Dulbecco's Modified Eagle Medium (DMEM). Aggregates were dislodged by gentle pipetting, and debris was removed by passing the suspension through a 70-μm cell strainer. Isolated cells were counted and plated on a Nunc™ Non-Treated 6 well plate (Thermofischer Scientific) at 1x10^6 cells/ml in DMEM glutamax, high glucose, high pyruvate (Thermofischer Scientific) supplemented with 10% FCS, 1 % non-essential amino acids, 1% penicillin/streptomycin and 2μM Mercaptoethanol (Thermofischer Scientific). To induce macrophage formation 25ng/ml macrophage colony-stimulating factor (MCSF) (Biolegend, London, UK) was added to the culture medium. Cells where then placed in a humidified incubator at 37ºC at 5% CO_2. Media was changed for fresh media every 48 hours and grown for 5 - 7 days.

In vitro macrophage stimulation: Cells were used at 1x10^6 cells per ml in DMEM glutamax, high glucose, high pyruvate (Thermofischer Scientific) supplemented with 10% FCS, 1 % non-essential amino acids, 1% penicillin/streptomycin and 2µM Mercaptoethanol, plated in 12 or 24 well plates. For titration experiments cells were exposed to increasing concentrations of LPS or IFNγ. For full M1 polarisation cells were stimulated with 100ng/ml LPS (from Escherichia coli O55:B5-Sigma) and 50ng/ml IFNγ (Thermofischer Scientific), whereas canonical M2 polarisation was achieved by 25ng/ml IL-4 (BD Biosciences, Berkshire, UK). In some experiments, cells were primed with various concentrations of thrombin (Enzyme Research Lab, Swansea UK) for 24 hours prior to exposure to IFNγ, LPS or IL-4. In some experiments, thrombin primed cells were incubated for 1 hour with anti IFNγ (ab9657 Abcam) prior to LPS stimulation. To asses the role of EPCR signalling cells were incubated for 2 hours with a neutralising ePCR antibody CD201 (EPCR) Monoclonal Antibody (eBio1560 (1560) Thermofischer Scientific) prior to thrombin stimulation. All experiments occurred in 10% FCS containing media.
ELISA: IFNγ and IL-10 ELISA Kits were purchased from ThermoFischer Scientific. Cell culture supernatants, reagents and standards were prepared as per manufacturer's instructions. Plates were read using a *SpectraMax® Plus 384* Microplate Reader (Molecular Devices) using 450nm as the primary wavelength and 620nm as reference wavelength. Data was analysed using SoftMax® Pro Software (Molecular Devices) and Excel software (Microsoft).
Flow Cytometry: All flow cytometry was performed on a Fortessa LSR II flow cytometer (Becton Dickinson) using DIVA software (Becton Dickinson) and analysed using Flow-jo (Treestar, Ashland, OR) software. Prior to surface staining cells were incubated with mouse Fc Block (Biolegend) for 5 minutes in the dark at 4°C, after which 50µL of the relevant antibody cocktail was added, and the cells were left to incubate in the dark at 4°C for 30 minutes. Surface antibodies were FITC – F4/80, APC- CD11b, PE-ABCA1 (Santa Cruz Biotechnology, Heidelberg, Germany). After surface staining cells were resuspended in 200µl pre-diluted Near IR live/dead stain (Life Technologies) and left to incubate in the dark at 4°C for 15 minutes. For intracellular staining, cells were permeabilised with Foxp3 intracellular staining permeabilisation solution for 30 minutes (e-Bioscience). Intracellular staining was performed using directly conjugated antibodies (BV605-CD206 (Biolegend) and PE-Cy7-iNOS (e-Bioscience)) made up into a staining cocktail using permeabilisation buffer (e-Bioscience), 50µL of staining cocktail was added per well and staining took place at 21 °C in the dark.
Lipid raft staining: Lipid rafts were stained using Vybrant™ Alexa Fluor™ 488 Lipid Raft Labeling Kit from Thermofischer scientific. Cells were plated on 8-well borosillicate IF wells (Nunc LabTek) at 1x10^5 cells/well in 200µl DMEM. 50U/ml Thrombin or MCSF was added to the wells and the cells were left overnight in an incubator at 37°C at 5% CO_2. The following day, after two washes with cold, serum-free DMEM, cells were incubated with 200µl CT-B-Alexa488 (1:1000 in serum-free DMEM for 10 minutes in the dark at 4°C. Following further washes with IF buffer containing PBS + 5% FCS + 10mM glycine, cells were incubated at RT for 2 hours with 500µl of 1:200 Rabbit αCT-B in serum free DMEM. For co-staining the primary antibody (Anti-TLR4 (ab13867) or IFN-γRβ Antibody (Santa Cruz)) was added to the Rabbit anti-CT-B for the first hour, the wells were then washed and for the second hour the secondary (goat anti-rabbit AF594 or goat anti-hamster AF594 (Thermofischer scientific)) was added to the Rabbit anti-CT-B. After a final washing step with IF buffer cells were fixed with 4% paraformaldehyde (PFA) for 10 minutes. Nuclear staining was undertaken with DAPI. Images were acquired using Image using an inverted confocal microscope @ 60x magnification (oil immersion) and analyzed using NIS-Elements software.
SiRNA: BMM were plated at 2x10^5 cells/ml in DMEM 10% FCS. 500 µL cell suspension was added to a 24 well plate. SiRNA was prepared using Lipofectamine™ RNAiMAX Transfection Reagent with Opti-MEM™ Reduced Serum Medium and Silencer™ Pre-Designed siRNA (ThermoFisher Scientific). Cells were transfected in complete medium for 24 hours as per manufacturers instruction at 37°C at 5% CO2. FITC conjugated positive control SIRNA and negative control SIRNA was also used (sc-36869 and sc-37007 respectively, Santa Cruz). After 24 hours 50U/ml thrombin was added, and cells were further incubated for 24 hours at 37°C at 5% CO_2. After this 24-hour incubation the cells were then washed and new media with LPS/IFNγ with or without thrombin was added for a further 24 hours. Cells were then analysed for ABCA1 and iNOS expression using flow cytometry as described above.
Western blot: Cells were removed from the tissue culture plate with 150 µL RIPA buffer (Abcam). Protein quantification was performed using the Pierce™ BCA Protein Assay Kit (Thermo Scientific). The XCell SurelockTM Mini-Cell (Invitrogen, California) was set up using pre-cast gels 4-12% (Biorad) and PageRuler™ Plus Pre stained Protein Ladder (Thermofisher) was used as control. The gel was set to run at 200v for 35 minutes. The XCell II blot module (Invitrogen, California) was used to transfer proteins from

the gel to a nitrocellulose membrane (Biorad) The transfer was run for 1 hour at 30v. The membrane was blocked for 1 hour in 5% powdered milk w/v (Marvel, UK) in TBS + 0.1% Tween-20 (TBST). Primary antibody Anti-ABCA1 antibody (ab7360) (Abcam) was added at 1/500 and anti-rabbit GAPDH (1/10000) in 5% milk in TBST overnight at 4°C. The secondary antibody (Anti Rb IRDye® 800CW, Li-Cor) was added at 1:10000 in TBST with 1% BSA for 1 hour at room temperature. Images were acquired using Odyssey® Fc Imaging System and analysed using Image Studio Lite (both Li-cor).

RT PCR: RNA was extracted using RNeasy mini Kit (Qiagen). RNA quantity was analysed by Nano drop system. Reverse transcription was carried out using the QuantiTect Reverse transcription Kit (Qiagen). Genomic DNA was eliminated using the provided gDNA wipe-out buffer. The PCR step was performed using TaqMan fast advanced master mix with TaqMan gene expression assays (ThermoFisher scientific). PCR assays used were: TBP (Mm01277042_m1), iNOS (Mm00440502_m1), TNFα (Mm00443258_m1), IL-1β (Mm00434228_m1), IL-6 (Mm00446190_m1) and RANTES (Mm01302427_m1). The plate was then set up on the BioRad CFX96 Real Time PCR detection system. The results were then compared to the housekeeping gene TATA box binding protein (TBP).

Statistical analysis: All statistical analyses were performed using GraphPad Prism® software version 7. Unpaired samples were compared using a Mann Whitney U test with two tailed p-values. P-values are shown as *P ≤ 0.05, ** P ≤ 0.01, ***P ≤ 0.001, **** P ≤ 0.0001.

Supplemental references

CHEN, D., GIANNOPOULOS, K., SHIELS, P. G., WEBSTER, Z., MCVEY, J. H., KEMBALL-COOK, G., TUDDENHAM, E., MOORE, M., LECHLER, R. & DORLING, A. 2004. Inhibition of intravascular thrombosis in murine endotoxemia by targeted expression of hirudin and tissue factor pathway inhibitor analogs to activated endothelium. *Blood,* 104**,** 1344-9.
